# Metastatic Prostate Cancer Mimicking Polymyalgia Rheumatica

**DOI:** 10.1155/2011/695320

**Published:** 2011-12-15

**Authors:** Charles T. Randazzo, Aaron W. Bernard, Douglas A. Rund

**Affiliations:** Department of Emergency Medicine, The Ohio State University Medical Center, 4833 Cramblett Hall, 456 W. 10th Avenue, Columbus, OH 43210, USA

## Abstract

A 59-year-old male presented to the emergency department with a four-month progressive history of proximal muscle pain and weakness with elevated erythrocyte sedimentation rate and C-reactive protein. He was initially diagnosed with polymyalgia rheumatica (PMR) and admitted to the hospital. During his hospitalization he was found to have metastatic prostate cancer, which was thought to be responsible for his PMR-like syndrome. By recognizing the resemblance between metastatic malignancy and rheumatologic diseases, the emergency physician can improve diagnostic accuracy.

## 1. Introduction

Polymyalgia rheumatica (PMR), the most common inflammatory rheumatic disease of the elderly, is characterized by proximal muscle pain and stiffness with an elevated erythrocyte sedimentation rate (ESR) and C-reactive protein (CRP) [1, 2]. The diagnosis, however, is not always straightforward [3]. Challenges in diagnosis arise from the heterogeneity of presentation with frequent atypical symptoms [3, 4], the large differential diagnosis of conditions that mimic PMR [2, 4–6], and a lack of agreed upon diagnostic criteria [1, 7–10]. When a patient presents to the emergency department with a PMR-like syndrome, the goal is to identify any potentially serious or life-threatening conditions responsible. Therefore, the emergency physician must be aware that many disease processes resemble PMR, some of which will require urgent intervention or admission to the hospital for further workup.

## 2. Case Presentation

A 59-year-old male with no known past medical history presented to the emergency department (ED) with diffuse, intermittent pain, and subjective fevers for four months. The pain was most prominent in the proximal muscle groups, notably in the hips, shoulders, neck, and lower back. The symptoms started four months prior with subjective fevers and intermittent muscle aches and progressed to include daily fevers with sweats, worsening pain, weakness, fatigue, and dyspnea. He reported making three other trips to the ED over the subsequent months, where he was repeatedly found to have elevated erythrocyte sedimentation rate (ESR) and C-reactive protein (CRP). He was treated with pain medication and scheduled for outpatient rheumatology followup. He was seen by rheumatology for the first time the day of the current presentation and was noted to appear ill. He was immediately directed back to the ED for further evaluation. On review of systems in the ED, he denied temporal pain, vision changes, or urinary complaints. 

His vital signs upon arrival to the ED were: temperature 98.5 degrees Fahrenheit, blood pressure 135/77 mmHg, pulse rate 102 beats/min, respiratory rate 20 breaths/min, and oxygen saturation 99% on room air. Physical exam was remarkable for a morbidly obese, uncomfortable patient, with tenderness to palpation of the muscles especially at the limb girdles and generalized weakness possibly related to pain. Strength was 4/5 globally, and range of motion was diminished in all extremities, but sensation and reflexes were intact. His HEENT exam was notable for a lack of focal tenderness over the temples. The remainder of his physical examination was within normal limits.

Cardiac workup was negative and chest X-ray showed no acute process. Blood cultures were sent, which would later show no growth. Pertinent laboratory values included Hemoglobin of 8.4 g/dL (normal range 13.2–17.3 g/dL), ESR > 140 mm/hr (normal range 0–19 mm/hr), CRP > 380 mg/L (normal range < 10 mg/L), ALT of 48 U/L (normal range 10–40 U/L), AST of 56 U/L (normal range 5–34 U/L), Alkaline phosphatase of 2285 U/L (normal range 38–126 U/L), and Creatine kinase of 175 U/L (normal range 37–174 U/L).

He was initially diagnosed with polymyalgia rheumatica and treated in the ED with pain medication and stress-dose steroids, and admitted for further workup. During his hospitalization, he was followed by rheumatology and treated with Prednisone 60 mg PO daily. A right upper quadrant ultrasound showed several nonspecific liver lesions, and in the context of his overall presentation, markedly elevated alkaline phosphatase and mildly elevated transaminases, a search for underlying malignancy was undertaken. CT scans of the chest, abdomen, and pelvis revealed an enlarged prostate and extensive bony lesions as well as pulmonary and liver nodules concerning for metastatic malignancy. PSA was elevated at 7.47 ng/mL (normal range < 4.00 ng/mL). Prednisone was tapered during his hospitalization. His symptoms were felt to be a PMR-like syndrome secondary to metastatic prostate cancer. Later, a bone scan was positive for diffuse osteoblastic metastatic disease, and a prostate biopsy revealed adenocarcinoma. He was discharged in stable condition, and chemotherapy was later initiated.

## 3. Discussion

This is a case of metastatic prostate cancer presenting with signs and symptoms that resemble PMR. Recent guidelines recommend a stepwise approach to diagnosis of PMR, involving evaluation of core inclusion criteria, core exclusion criteria, and assessing response to steroids [1]. Core inclusion criteria include age > 50, duration > 2 weeks, bilateral shoulder and/or pelvic girdle pain, morning stiffness, and evidence of acute-phase response [1]. These criteria were all met in our patient, perhaps with the exception of morning stiffness. Core exclusion criteria are active infection, active malignancy, and active giant-cell arteritis (GCA) [1]. 

Atypical features of PMR in our patient included weakness, very high ESR and CRP, markedly elevated alkaline phosphatase, prominent systemic symptoms, duration > 2 months, and age < 60 [1]. However, systemic symptoms such as fever, malaise, and fatigue are present in 40% of patients with PMR [2]. Additionally, while weakness is typically considered absent in PMR, difficulty distinguishing true weakness from reluctance to move a joint due to pain has been described in this disease, as well as mild weakness from disuse atrophy [3]. ESR < 40 or > 100 is considered atypical [1], but alkaline phosphatase can be elevated [11].

PMR as the presentation of metastatic malignancy is well known but relatively rare [12]. Prior cases of metastatic prostate cancer mimicking PMR have been reported [13]. Other malignancies reported to mimic PMR include solid tumors of the kidney, stomach, colon, lungs, pancreas, uterus and ovaries, and hematologic malignancies such as multiple myeloma and lymphoma [4, 5, 14]. Rheumatologic syndromes in the context of cancer can be due to a paraneoplastic syndrome or metastatic involvement of joints. However, immune dysregulation, which can cause a rheumatologic disease first, may lead to malignant transformation later in the disease course [15]. 

Many argue that a search for occult malignancy is unnecessary in classic presentations of PMR but required when there are atypical features [5, 12, 13, 15, 16]. As demonstrated in our case report, advanced imaging and tumor markers can be helpful in these situations. Specifically regarding prostate cancer, some have argued for a digital rectal exam or PSA in all male patients with suspected PMR, especially those with atypical features [13]. 

In addition to malignancy, other rheumatologic diseases are commonly mistaken for PMR [4–6]. These include rheumatoid arthritis, RS3PE syndrome, systemic lupus erythematosus, spondyloarthropathy, inflammatory myopathy, calcium pyrophosphate deposition disease, and vasculitides [4, 5]. Nonrheumatologic diseases that may resemble PMR including infective endocarditis, hypothyroidism, hyperparathyroidism, cervical stenosis, Parkinson's disease, statin-induced myalgia, and fibromyalgia [2, 4–6]. 

Clues to a diagnosis other than PMR should be sought from the history, physical exam, diagnostic studies, and clinical course. These include lack of pain exacerbation with movement, diffuse aching, minimal morning stiffness, high fever, distal arthritis, organ enlargement, painless adenopathy, cytopenia, immature blood cells, monoclonal gammopathy, transaminitis, elevated muscle enzymes, hematuria, and poor response to steroids [1, 4]. In a patient recently diagnosed with PMR, failure of steroid therapy may signify GCA, malignancy, infective endocarditis, spondyloarthropathy, or amyloidosis [5]. Other clues that may specifically suggest malignancy include personal or family history of malignancy, carcinogen exposure, coexistent paraneoplastic syndromes, and serologic tumor markers [15].

The occasional resemblance between malignancy and PMR poses a challenge not only for the diagnosis of malignancy but also for the diagnosis of PMR. Cases have been reported in which patients with known prostate cancer develop superimposed PMR, but the symptoms are initially attributed to the cancer, obscuring the diagnosis of PMR and delaying treatment [11].

The emergency physician should be aware of the aforementioned conditions that resemble PMR but must also evaluate for GCA, a serious condition within the same disease spectrum of PMR. Evidence of GCA in a patient with suspected PMR include abrupt onset temporal headache, temporal tenderness on exam, vision changes, jaw or tongue claudication, prominence or beading of the temporal artery, diminished temporal pulse, cranial nerve palsies, and limb claudication [1]. If GCA is suspected, steroids must be administered immediately, as delay can lead to blindness. However, in suspected PMR without evidence of GCA, steroid therapy can wait until the diagnosis of PMR is established, and a search for other diseases such as malignancy is initiated, if necessary [1].

Our patient reported a four-month history of progressive symptoms with multiple ED visits prior to the current presentation, as he waited for outpatient rheumatology evaluation. Details of his earlier presentations were not fully known, but it could be speculated that clues to his true diagnosis were missed. By evaluating patients with a PMR-like syndrome for atypical features, the emergency physician has the power to facilitate earlier detection of serious conditions such as GCA and metastatic malignancy.
